# Clinical Outcome After Microsurgical Resection of Central Neurocytoma: A Single-Centre Analysis of 15 Years

**DOI:** 10.3389/fneur.2021.790641

**Published:** 2021-12-22

**Authors:** Dan Cao, Yong Chen, Zhengqian Guo, Yibo Ou, Jian Chen

**Affiliations:** Department of Neurosurgery, Tongji Medical College, Tongji Hospital, Huazhong University of Science and Technology, Wuhan, China

**Keywords:** central neurocytoma, neurosurgery, complication, outcome, the extent of resection

## Abstract

**Objective:** This study aimed to explore the immediate postoperative and long-term outcomes of central neurocytoma (CN) based on 15 years of experience in our institution.

**Methods:** This single-institution study collected data of 43 patients with CN who underwent surgery between 2005 and 2020. We reviewed data of clinical, immediate postoperative outcome, and long-term outcome of patients. More specifically, we divided complications into neurological and regional complications groups.

**Results:** Among the 43 patients with CN who underwent surgery, the transcortical (72.1%) or transcallosal (25.6%) approach was used. There were 18 patients (41.9%) who complained about postoperative neurological complications, including motor weakness (25.6%), memory deficit (18.6%), aphasia (7.0%), and seizure (4.7%). In addition, 18 patients suffered postoperative regional complications such as hydrocephalus (2.3%), hematoma (34.9%), infection (4.7%), and subcutaneous hydrops (2.3%). Only one-quarter of patients had suffered permanent surgical complications. The majority of patients recovered from the deficit and could turn back to normal life. There were no significant differences in the clinical outcomes between transcortical and transcallosal approaches. At a median follow-up of 61.8 months, the 5-year overall survival and progression-free survival were 87.0 and 74.0%, respectively. A multivariate Cox model analysis showed that the extent of resection was not related to progression-free survival. However, the extent of resection was significantly associated with overall survival, and gross total resection decreased the risk of death.

**Conclusions:** Patients with CN show favorable outcomes after surgery. The transcortical and transcallosal approaches have similar postoperative complication rates and long-term follow-up outcomes. In terms of long-term prognosis, maximal safety resection should be the first choice of CN.

## Introduction

Central neurocytoma (CN) is a rare neoplasm and accounts for 0.01–0.50% of all intracranial tumors (1–3). CN was first recognized as a distinct ventricular tumor by Hassoun et al. (4), with features of a benign clinical behavior and neuronal differentiation in 1982. CN has become an autocephalous entity based on clinical and pathological characteristics afterward. Extraventricular neurocytoma (EVT) has the same histopathological characteristics as CN, but it is located outside the ventricle (5). CN and EVT are grade II intracranial tumors, according to the 2007 World Health Organization Classification of tumors of the central nervous system (6).

Central neurocytoma tends to affect young adults in the 20–34 years range. Also it does not show sex preponderance (1, 7, 8). As it is typically located in a deep midline position near the foramen of Monro and septum pellucidum, they often present with symptoms and signs of obstructive hydrocephalus such as headache, nausea, vomiting, papilledema, and seizures.

Surgery remains the primary treatment strategy for CN. The surgical management of CN represents a formidable challenge due to deep eloquent overlying neurovascular structures surrounded by the tumors. There are some surgical approaches for CN in the ventricle depending on the location, size, or attachment of the tumor, including transcallosal and transcortical approaches. It is indisputable that the therapeutic schedule for CN should focus on tumor control and also on the quality of life because CN usually occurs in young adults and has an indolent clinical process.

Due to of the rarity of this tumor, there are limited studies that mentioned surgical complications and mortality of CN. Our analysis of information on 43 patients with CN is intended to provide new data that will support surgical decisions. This work aims to review our experience with an emphasis on surgical complications as well as outcomes.

## Methods

### Clinical Features

Forty-three consecutive CN cases diagnosed between January 2005 and October 2020 were included. Six EVT cases and three recurrent CN cases, in which the initial surgery was not performed in our hospital, were identified but excluded from this study. Data regarding patients' general information, details of the surgery, histopathologic features, surgical complications, and outcomes were obtained retrospectively.

### Operative and Postoperative Data

All patients underwent craniotomy in our institution. Surgical records and postoperative radiologic images were reviewed. Follow-up data were acquired from outpatient department visits and telephone interviews. The extent of resection (EOR) of surgical resection was classified as gross total resection (GTR) or non-GTR. GTR has been defined as no residual tumor in postoperative images or/and 100% macroscopic resection of the tumor. Besides, we defined it as non-GTR.

More specifically, we categorized the clinical outcomes of the patients as flows: postoperative KPS, neurologic complications (motor weakness, memory deficit, aphasia, and seizure), regional complications (hydrocephalus, hematoma, infection, and subcutaneous hydrops), and others.

Long-term outcomes included OS and PFS. The period of OS was defined as the time interval between the initial treatment and the date of death or last follow-up. The period of PFS was defined as the time between the initial treatment and the date of recurrence based on radiological findings.

### Pathology

The diagnosis of CN was confirmed by pathological examination according to the 2016 World Health Organization criteria. The tumor is composed of uniform small cells with round, regular nuclei, clear cytoplasm, spotted chromatin, and perinuclear halo (9). Immunohistochemically, it is usually positive for synaptophysin, neuron-specific nuclear protein (NeuN), glial fibrillary acidic protein, and neuron-specific enolase. Among them, NeuN shows higher specificity (10). Atypical central neurocytoma is a clinically aggressive variant of neurocytoma and harbors the following pathological characteristics: focal necrosis, vascular proliferation, nuclear atypia, infiltrative margins, and increased mitotic activity exhibiting ki-67 index >2% (11).

### Statistical Analysis

The analyses were performed using SPSS (IBM SPSS Statistics 23). Chi-squared test and Fisher's exact test were employed to analyze categorical variables. The multivariate logistic regression analysis was performed to calculate the prognostic value of the risk factors that result from complications immediately following surgery. Differences between survival curves were assessed using a log-rank test. For all tests, a *p*-value of < 0.05 was deemed to indicate statistical significance.

### Literature Review

We made a systematic literature search by the use of Medline (PubMed by the National Library of Medicine, National Institutes of Health, Bethesda, Maryland, USA) and used the following keywords: “central neurocytoma,” “transcortical,” “transcallosal,” which gave 13 results. The studies were selected using the Preferred Reporting Project for Systematic Reviews and Meta-Analysis (PRISMA) criteria. Only studies that included more than 10 patients were a part of our review. Meanwhile, each of the studies included transcallosal and transcortical approaches and surgery-related complications. At the same time, we limited the published year to between 2005 and 2021. We further read the references of included articles that met the criteria to find additional reports.

## Results

### Patients and Tumor Characteristics

The information including demographics, presenting symptoms, and tumor characteristics of 43 patients with CN were summarized in Table 1. Our series included 26 (60.5%) men and 17 (39.5%) women, with a mean age of 33.7 years (range, 12–57 years). Intracranial hypertension was the most common presenting symptom, including headache, nausea and vomiting, and blurred vision. Other symptoms included dizziness, weakness, seizure, and tinnitus. Several patients presented with multiple symptoms. Only 4 (9.3%) patients presented asymptomatic.

**Table 1:** Clinical, demographic, and pathological characteristics in 43 patients with central neurocytoma.

**Variable**	***N*** **(%) 43**
**Age**	
Mean/range	33.7 ± 10.92/12-57 years
**Gender**	
Male/female	26 (60.5)/17 (39.5)
**Symptoms**	
Headache	30 (69.8)
Dizziness	13 (30.2)
Nausea and vomiting	7 (16.3)
Blurred vision	4 (9.3)
Weakness	5 (11.6)
Seizure	3 (7.0)
Tinnitus	1 (2.3)
Asymptomatic	4 (9.3)
**Tumor location**	
Left lateral ventricle	18 (41.9)
Right lateral ventricle	15 (34.9)
Both lateral ventricle	9 (20.9)
Third ventricle	6 (14.0)
Fourth ventricle	1 (2.3)
Tetraventricle	1 (2.3)
**Tumor size**	IQR:36.50–60.00 mm
	Mean 46.72 ± 17.45 mm
	Media 44.50 mm
**Pathological features**	
**Ki-67 index**	IQR:2–5%
	Mean 5.7 ± 12.1%
	Media 3.0%
**IHC for SYN**	
Positive/negative/equivocal	38/2/3
**IHC for Neun**	
Positive/negative/equivocal	38/4/1
**IHC for Nestin**	
Positive/negative/equivocal	9/32/2
**IHC for GFAP**	
Positive/negative/equivocal	20/19/4

In terms of tumor location, CN was located most commonly in the lateral ventricle. There were six tumors that invaded the third ventricle, one invaded the fourth ventricle, and one involved tetraventricle. The maximum tumor diameter ranged from 10 to 86 mm, with a mean diameter of 46.72 mm. Four asymptomatic patients had a relatively small tumor diameter with a mean diameter of 38.00 mm. The pathological features were listed in Table 1. In our series, the mean ki-67 index was 4.0% (media, 3.0%; range, 1–25%).

### Treatments and Surgical Complications

Surgical approaches were divided into two types: transcortical and transcallosal. The transcortical approach was performed in 31 (72.1%) cases and was the most popular approach in our institution. The transcallosal approach was used for 11 (25.6%) patients. One patient (2.3%) underwent a modified pterional approach for a tetraventricular tumor. The GTR and non-GTR surgery were performed in 28 (65.1%) and 15 (34.9%) patients, respectively (Table 2). In the GTR group, postoperative radiation therapy (RT) was performed in 14 patients with atypical histological features. In the non-GTR group, postoperative RT was performed in 7 patients.

**Table 2:** Treatment characteristics in 43 patients with central neurocytoma.

**Treatment modalities**	***N*** **(%) 43**
**Approach**	
Transcortical	31 (72.1)
Transcallosal	11 (25.6)
Modified pterion	1 (2.3)
**Treatment details**	
Gross total resection	28 (65.1)
Surgery alone	10 (23.3)
Surgery and RT followed	14 (32.6)
Unknown	4 (9.3)
Non-gross total resection	15 (34.9)
Surgery alone	5 (11.6)
Surgery and RT followed	7 (16.3)
Unknown	3 (7.0)

**Figure 1:**
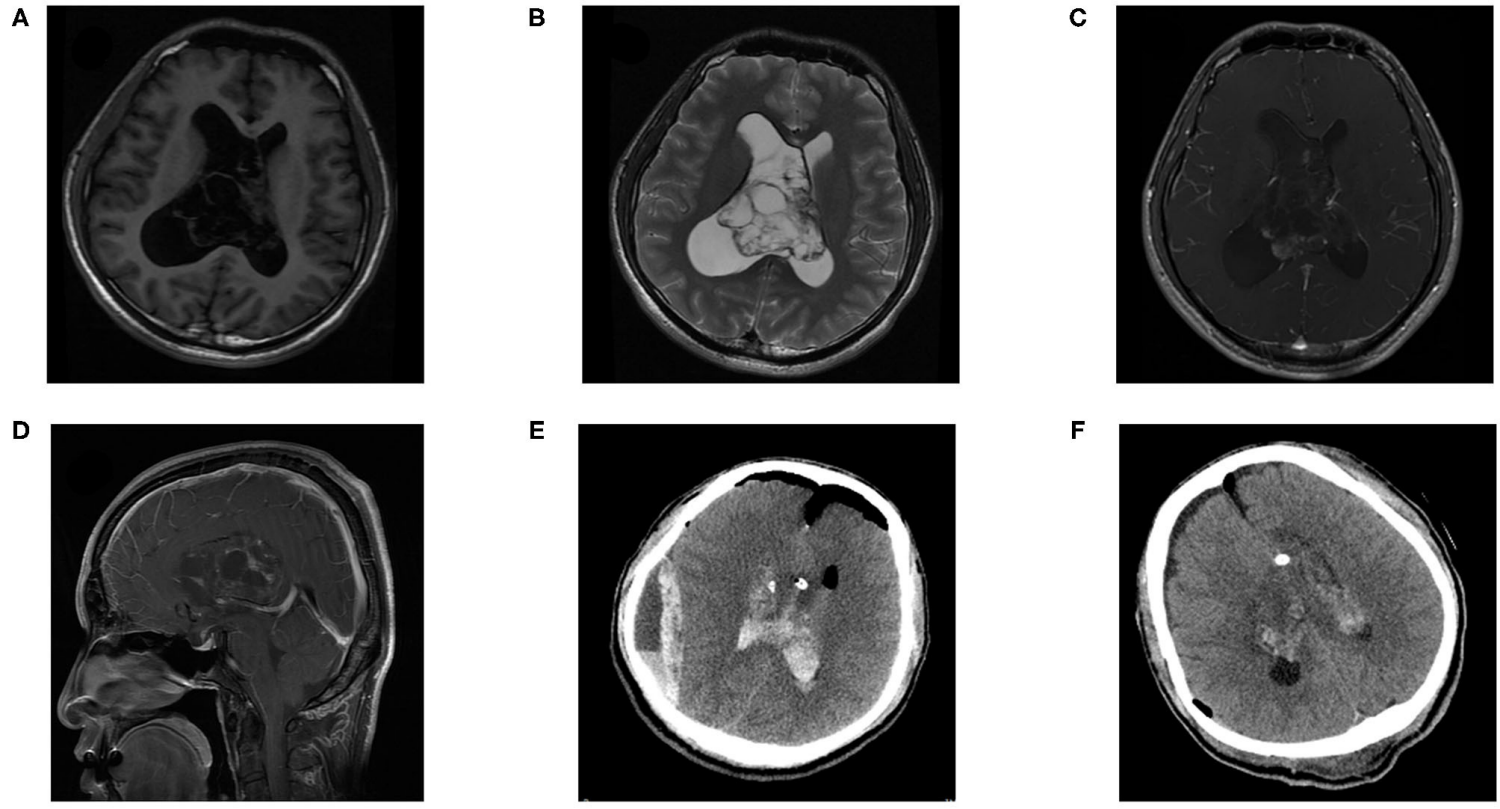
A 27-year-old female patient suffered epidural hematoma with central one day after surgery. Pre-operative **(A)** T1 weighted axial sequence, **(B)** T2-weighted axial sequence, **(C)** T1-enhanced axial sequence, **(D)** T1-enhanced sagittal sequence, MRI scans showing a 61 × 65 × 37 mm-sized mass in bilateral lateral ventricles, **(E)** CT revealed an epidural and cavity hematoma after the tumor resection. **(F)** CT showed that the hematoma had been cleared, indicated that there was residual tumor.

The postoperative complications and long-term outcomes of patients were summarized in Table 3. The overall rate of postoperative complication was 51.2%. Karnofsky performance status (KPS) score of 24 (55.8%) patients remained unchanged, 15 (34.9%) patients declined, and 4 (9.3%) patients improved. More specifically, 18 patients complained about neurological complications, including motor weakness, memory deficit, aphasia, or seizure. In addition, 18 patients suffered regional complications such as hydrocephalus, hematoma, infection, or subcutaneous effusion. In terms of hematoma, 10 patients experienced intraventricular hemorrhage, 4 patients suffered epidural hematoma (Figure 1), and 1 patient had a subdural hematoma. One patient who suffered gastrointestinal bleeding was recovered with medical treatment. Multiple complications could have occurred in a single patient.

**Table 3:** Postoperative complication and outcomes according to surgical approach.

**Postoperative presentation** **(***n =*** 43)**	**Postoperative (%)**			**Last follow-up (%)**		
	**Overall** **(***n =*** 43)**	**Transcortical** **(***n =*** 31)**	**Transcallosal** **(***n =*** 11)**	**Overall** **(***n =*** 36)**	**Transcortical** **(***n =*** 26)**	**Transcallosal** **(***n =*** 10)**
**Complication**	22 (51.2)			11 (30.6)		
**KPSS**	***n*** **= 42**			***n*** **= 36**		
KPSS unchanged	24 (55.8)	18 (58.1)	5 (45.5)	22 (61.1)	16 (61.5)	6 (60.0)
KPSS declined	15 (34.9)	10 (32.3)	5 (45.5)	10 (27.8)	7 (26.9)	3 (30.0)
KPSS improved	4 (9.3)	3 (9.7)	1 (9.1)	4 (11.1)	3 (11.5)	1 (10.0)
**Neurological complications**	***n*** **= 18**			***n*** **= 8**		
Motor weakness	11 (25.6)	8 (25.8)	3 (27.3)	5 (13.9)	3 (11.5)	2 (20.0)
Memory deficit	8 (18.6)	5 (16.1)	3 (27.3)	3 (8.3)	1 (3.8)	2 (20.0)
Aphasia	3 (7.0)	2 (6.5)	1 (9.1)	1 (2.8)	1 (3.8)	0 (0)
Seizure	2 (4.7)	1 (3.2)	1 (9.1)	0 (0)	0 (0)	0 (0)
**Regional complications**	***n*** **= 18**			***n*** **= 2**		
Hydrocephalus	1 (2.3)	1 (3.2)	0 (0)	2 (5.6)	1 (3.8)	1 (10.0)
Hematoma	15 (34.9)	10 (32.3)	4 (36.4)	0 (0)	0 (0)	0 (0)
Intraventricular	10 (23.3)	6 (19.4)	4 (36.4)	0 (0)	0 (0)	0 (0)
Epidural	4 (9.3)	3 (9.7)	1 (9.1)	0 (0)	0 (0)	0 (0)
Subdural	1 (2.3)	1 (3.2)	0 (0)	0 (0)	0 (0)	0 (0)
Infection	2 (4.7)	1 (3.2)	0 (0)	0 (0)	0 (0)	0 (0)
Subcutaneous hydrops	1 (2.3)	1 (3.2)	0 (0)	0 (0)	0 (0)	0 (0)
**Gastrointestinal bleeding**	1 (2.3)	0 (0)	1 (9.1)	0 (0)	0 (0)	0 (0)
**Death**	3 (7.0)	2 (6.5)	1 (9.1)	7 (19.5)	3 (11.5)	4 (40.0)

The majority of patients recovered from the deficit and could turn back to normal life. Only one-quarter of patients had suffered permanent surgical complications, including 8 neurological and 2 regional complications. There was no significant difference in EOR (*p* = 0.72) and clinical outcomes between the transcortical and transcallosal approaches. General information was listed in Table 4. More specifically, GTR was achieved in 20 of 31 (64.5%) patients with transcortical approach and in 8 of 11 (72.7%) patients who underwent transcallosal approach. There was no significant difference in clinical outcomes according to the surgical approaches, including mean length of hospital stay (*p* = 0.90), mean volume of blood loss (*p* = 0.70), KPS score changes at discharge (*p* = 0.16), KPS score changes at last follow-up (*p* = 1.00), hematoma (*p* = 0.71), and intraventricular hematoma (*p* = 0.22). In addition, we compared other clinical outcomes such as motor weakness (*p* = 1.00), memory deficit (*p* = 0.41) of the transcortical approach with the transcallosal approach and found that significant difference did not persist postoperatively or at the final follow-up. In Table 5, we concluded surgical clinical series which were published in English from 2010 to 2021. The surgical approaches and perioperative complications of the previous clinical series were listed.

**Table 4:** The extent of resection and clinical outcomes according to surgical approach.

	**Transcortical approach** **(***n =*** 31)**	**Transcallosal approach** **(***n =*** 11)**	***P***
**Extent of resection**			
GTR	20	8	0.72
Non-GTR	11	3	
**Outcomes**			
**Mean length of hospital stay(days)**	26.9	23.6	0.90
**Mean volume of blood loss (ml)**	867.4	1,134.4	0.70
**KPS at discharge**			
Favorable	22	5	0.16
Unfavorable	9	6	
**KPS at last follow-up**			
Favorable	19	7	1.00
Unfavorable	7	3	
**Motor weakness**			
No deficit	23	8	
Transient	8	3	1.00
Persistent	3	2	0.60
**Memory deficit**			
No deficit	26 (25)^※^	8	
Transient	5	3	0.41
Persistent	1	2	0.18
**Hematoma**			
Yes	10	4	0.71
No	21	6	
**Intraventricular hematoma**			
Yes	6	4	0.22
No	25	6	

*The KPS scores at the discharge were categorized as favorable if the KPS assessment was improved or unchanged, or unfavorable if it had worsened*.

※ *Twenty-six patients who underwent transcortical approach did not suffer memory deficit at discharge, twenty-five patients who underwent transcortical approach did not suffer memory deficit at the last follow-up*.

**Table 5:** Postoperative complications and outcomes of previous clinical series.

**Reference**	**Cases**	**Approach**	**Radical removal (%)**	**Mortality**	**30-day morbidity**					
					**Overall**	**Paresis**	**Hypomnesis**	**Rebleeding**	**Seizure**	**Hydrocephalus**
Hallock et al. (12)	19	–	52.6	5.3	15.8	–	–	5.3	–	–
Qian et al. (13)	92	TCO (52.2%) TCA (47.8%)	70.8 70.5	3.3 –	40.0 –	10.4 6.8	22.9 29.5	– –	4.2 6.8	58.3 65.9
Kim et al. (3)	58	TCO (39.6%) TCA (60.4%)	47.4 51.7	– –	– –	42.1 41.4	15.8 20.7	– –	36.8 10.3	– –
Lubrano et al. (8)	82	–	48.0	2.0	66.0	–	29.0	14.6	9.0	26.0
Chen et al. (14)	32	TCO (46.9%) TCA (53.1%)	73.3 76.5	– –	– –	6.7 5.9	6.7 17.6	– –	13.3 5.9	13.3 5.9
Wang et al. (15)	63	TCO (61.9%) TCA (38.1%)	53.9 54.1	7.7 8.3	– –	41.0 33.3	23.1 33.3	7.7 33.3	12.8 8.3	35.9 45.9
Byun et al. (16)	40	TCO (72.5%) TCA (22.5%)	62.5	5.0	50.0	30.0	2.5	–	–	–
Han et al. (17)	67	TCO (92.7%) TCA (7.5%)	82.1	–	–	32.8	–	9.0	–	43.3

*TCO, transcortical; TCA, transcallosal; –, not described*.

### Long-Term Follow-Up

**Figure 2:**
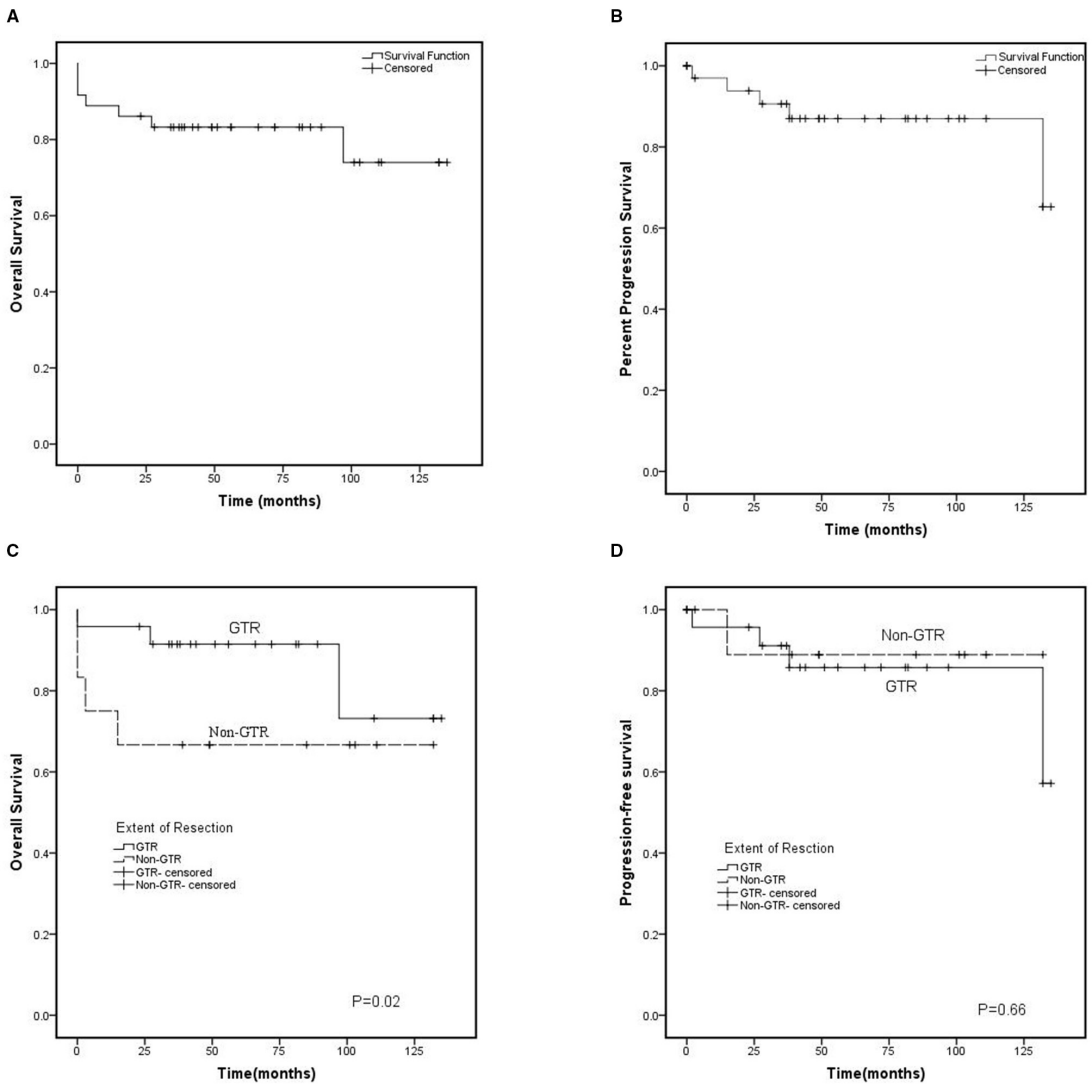
Kaplan-Meier curve. **(A)** Overall survival (OS) and **(B)** cl (PFS) for 36 patients with central neurocytoma between 2010 and 2020. **(C)** Overall survival and **(D)** Progression-free survival based on the initial extend of resection. GTR, gross total resection; Non-GTR-censored, non-gross total resection.

There were 36 patients who had adequate follow-up data for 0–135 months (mean, 61.8 ± 40.4 months). The 5-year PFS and OS rates were 87.0% and 74.0%, respectively (Figure 2). Five patients had experienced recurrence, of whom four patients had suffered tumor recurrence with ki-67 index >2. Univariate analysis of patient, tumor, and treatment prognostic factors on survival outcomes showed that none of them was associated with an increased risk of recurrence. Delayed recurrence (>5 years after initial diagnosis) occurred in 2 patients.

Seven patients died by the time of analysis, of whom 3 patients died due to serve postoperative complications, and 2 patients died because of tumor recurrence. These patients included 3 who underwent GTR, 2 who underwent non-GTR, and 2 who underwent non-GTR accompanied by RT. The other death was due to pulmonary infection and unknown reasons. Age, tumor size, tumor location, ki-67 index, EOR, and postoperative RT were assessed with mortality. Multivariate Cox model analysis showed that EOR was significantly associated with OS, and the GTR decreased the risk of death (Figure 2). Meanwhile, none of the other factors impacted mortality.

## Discussion

### Clinical Features

Central neurocytoma is an uncommon intraventricular tumor and thus, the number of clinical series is relatively limited in the literature. CN represents 0.01–0.50% of all primary brain tumors (1–3, 18, 19). It tends to affect young adults and is most common in their third decade (1, 19). Generally, previous literature has reported that CN is equally prevalent in either sex (7, 8). We found that the mean age was 33.7 years, which corresponded with the previous study, and noted a predominance of men with a man/woman ratio being 1.5:1 in our series.

Being initially “silent,” CN grows slowly and has a long clinical course. Symptoms are usually related to subsequent intracranial hypertension caused by the cerebrospinal fluid flow obstruction when it reaches a large size. The most common symptoms reported of CN are headache, nausea and vomiting, blurred vision, and also dizziness, seizure (Table 1).

### Surgical Approaches

In the previous literature, it is known that the primary treatment of CN is surgical resection (13, 15, 17); meanwhile, surgery remains challenging due to its intimate relation to deep eloquent critical structures. The traditional surgical approach for intraventricular CN includes the transcortical approach and transcallosal approach (13, 15). We should take the minimal damage to brain structures, best and safest exposing the vital structures, and expand the working angle into consideration when neurosurgeons select a route to intraventricular CN. Meanwhile, tumor size, its adjacent critical structures, and the neurosurgeon's preference also influence the choice of approach.

If the tumor is located in the body of one side of the ventricle, or both sides of the ventricle, or the third ventricle, the transcallosal approach offers short access to the tumor and flexibility of exposure with constant anatomy during the approach. In contrast, the transcortical approach is suitable for the tumor that originates from one side, especially in the anterior part of the lateral ventricle, and is ideal for patients with large ventricles.

The transcortical approach offers easy access to lateral ventricle and overview, but it may influence cortical intact. This route is associated with a high incidence of seizures and other related neurological deficits. In contrast, the transcallosal approach offers a shorter pathway to the third ventricle, although it may injure the fornix, parasagittal vein, pericallosal artery, or interhemispheric dissection. This approach may entail a higher risk of disconnection syndrome. GTR was achieved in 64.5% of transcortical and 72.7% of transcallosal approaches, respectively. There was no significant difference in EOR according to the approaches. In our center, there was also no significant difference in neurologic and regional complications between the two approaches.

The tubular retractor has been recently introduced into clinical practice and it has been found to be useful for ventricular or periventricular tumors (20–23). Although this valuable alternative technique is not widely described, we did not have the experience to perform tubular retractor in our series. However, the approaches performed in our series allowed access to the ventricular cavity through a small corticectomy in a non-eloquent area to minimize the damage to the cortex and brain tissue, and yet, the rate of our complications was the same as the previous literature (13, 14, 17, 23).

### Surgical Complications and Outcomes

There are limited studies that mentioned the surgical complications and mortality of CN (3, 13–16). In our series, the most common complications were motor weakness, hematoma, memory deficit, and cognitive complication. Fewer patients suffered seizure, speech disturbance, hydrocephalus infection, subcutaneous hydrops, etc. The probability of hydrocephalus was reduced with the improvement of surgery strategy, such as septostomy of the septum pellucidum.

Motor weakness ranged from 5.9 to 42.1% in previous studies (3, 14–16). We reported a 14.0% incidence of transient motor weakness. Operating on a ventricle tumor might require manipulation of motor control, retraction of a supplementary motor area, or sacrificing of bridging vein, which may result in motor weakness.

The previous study recorded memory deficit ranging from 2.5 to 33.3% (3, 13–16). Here there was up to 27.3% of transcallosal approaches and <16.2% memory deficit incidence of transcortical approaches in our series. Memory deficit is most probably caused by injury to the Papez memory circuit, including the corpus callosum and fornix or hydrocephalus. Chen and his colleague emphasized that the length of dissected corpus callosum should be <3 cm (14). Our manipulation corresponded to the former.

The intracranial hematoma is the common but urgent perioperative complication, including intraventricular hemorrhage (IVH), epidural hematoma (EDH), and subdural hematoma.

The finding from the previous survey reminded 5.0–9.5% incidence of EDH (17), which appeared in 4 patients (9.3%) of our series. The current consensus is that EDH is related to inappropriate maneuvers. It is commonly accepted that sudden reduced intracranial pressure at the time of craniotomy, cerebrospinal fluid release, or tumor removal may increase the dural venous transmural pressure, thus causing traction on the dural bridging veins. This ruptured blood vessel in turn strips the dura from the inner skull plate to form the hematoma. Ma et al. (24) have postulated that severe fluctuation of systemic blood pressure under general anesthesia may also be responsible for extension intracranial pressure fluctuation. Some scholars believe that we should take coagulation abnormalities into consideration (25, 26).

Intraventricular hemorrhage has been reported within a range of 5–25% risk (12, 15). Here, 23.3% of our patients experienced hemorrhage in the tumor cavity. We consider that incomplete hemostasis of surgery or coagulation dysfunction may result in IVH. In the 10 cases with intraventricular hemorrhage, the tumor size was more than 4 cm, and the blood loss was mostly more than 800 ml. Ma et al. (24) suggested that the cavitronultrasonic-surgical-aspirator can be quite helpful to control blood loss during tumor resection. Chaves et al. (27) raised that preoperative adjuvant embolization is a feasible strategy for the treatment of large CN. Some scholars believe that irrigation with saline after tumor resection is a useful way to remove the blood of the tumor cavity and make sure of satisfactory hemostasis (24). Apart from these factors, in our cases, temporary postoperative external ventricular drainage was routinely placed for 2–7 days after surgery. It helps drain blood out of the ventricle and minimizes the risk of postoperative hydrocephalus. More specifically, we suggest shutting off the drainage after the cerebrospinal fluid was cleared, and then removing the drainage if the patient is in good condition.

Reports stated a mortality rate of 0–8.3% (13–15). In all, three patients (7.0%) died within 30-day in our series. The causes for 30-day mortality were related to related treatments or tumors themselves, including secondary hemorrhage, brain swelling, and hydrocephalus (8).

### Survival Analysis

Most literature shows that patients with CN who underwent complete resection prefer to have a better prognosis. Complete resection can provide long-term local control and longer OS (12, 17, 18, 28). Nevertheless, some studies indicate that GTR provides long-term local control but does not significantly associate with longer OS (3, 8, 13). Several investigators found that the PFS rate was not statistically different between GTR and subtotal resection groups (15, 16). Nevertheless, GTR decreased the risk of death but does not prolong the PFS in our series.

There is no consensus whether postoperative adjuvant RT is effective in local control and potentially survival. Rades and Fehlauer (29) found that postoperative RT could improve local control and survival in the GTR group by a meta analysis of 310 patients. However, the largest study as yet showed that the postoperative RT was not statistically significant in potential survival by the analysis of 868 patients in the National Cancer Database (NCDB) (7). In our cohort, postoperative RT also did not improve local control and survival.

### Limitations

Several limitations warrant discussion. First, the data of our study were collected from medical records retrospectively, which may have caused bias. Second, during the past 15 years covering our study, the understanding of CN and the development of the techniques of microsurgical and endoscopic techniques have become advanced. Furthermore, given the rarity of CN, prospective and multi-institutional future studies are required to reveal the clinical outcomes for patients with CN.

## Conclusion

Central neurocytoma is a rare intraventricular tumor that usually affects young adults and has a predominance of men. Patients generally show favorable outcomes after microsurgical resection of CN. The transcortical and transcallosal approaches have similar complication rates and clinical outcomes. To protect function as much as possible, maximal safe resection should be the first choice of CN. Postoperative RT also does not improve local control and survival.

## Data Availability Statement

The original contributions presented in the study are included in the article/supplementary material, further inquiries can be directed to the corresponding authors.

## Author Contributions

DC: conceptualization, methodology, and writing–original draft. YC and ZG: date curation and software. YO: writing–review and editing. JC: supervision. All authors contributed to the article and approved the submitted version.

## Conflict of Interest

The authors declare that the research was conducted in the absence of any commercial or financial relationships that could be construed as a potential conflict of interest.

## Publisher's Note

All claims expressed in this article are solely those of the authors and do not necessarily represent those of their affiliated organizations, or those of the publisher, the editors and the reviewers. Any product that may be evaluated in this article, or claim that may be made by its manufacturer, is not guaranteed or endorsed by the publisher.
